# How to Grow a Professional Identity: Philosophical Gardening in the Field of Medical Education

**DOI:** 10.5334/pme.367

**Published:** 2023-01-06

**Authors:** Mario Veen, Anne de la Croix

**Affiliations:** 1 Department of General Practice, Erasmus University Medical Center, Rotterdam, NL; 2 Amsterdam UMC location Vrije Universiteit Amsterdam, Research in Education, Boelelaan 1118, Amsterdam, The Netherlands; 3 LEARN! Research Institute for Learning and Education, Faculty of Psychology and Education, Vrije Universiteit Amsterdam, Amsterdam, NL

## Abstract

In this philosophical reflection, we – following the philosopher Heidegger - introduce two farmers who represent different ways in which one can develop growth (see https://www.youtube.com/watch?v=g7jZigyfKHI for instructional video). One is a traditional farmer who entrusts the seeds to the soil and cultivates them with care and trust. The other is a modern farmer who takes an industrialized approach and positions the seeds and ‘challenges-forth’ the crops to show themselves in a certain way. We use these farmers as an analogy for the ways in which we as medical educators can and should relate to those we ‘care’ for: medical students and trainees.

Taking a philosophical stance, and accounting for our own positionality and involvement in the analysis, we focus on ‘Professional Identity Formation’ and its operationalization in the field of medical education. We identify three main approaches medical education has taken to identity: as an individual trait, as a set of behaviors, and as a socialization process. All these approaches have at their root a similar assumption, namely that all inner processes can be made visible. We challenge this representational paradigm and use ‘philosophical gardening’ to raise awareness of what can and cannot be measured and controlled.

Finally, we suggest educational approaches that leave space for diversity in students’ experiences, learning approaches, and growth. We share good practices of brave teachers and curriculum designers whose interventions are characterized by less control and fewer measurements of personal growth, but more trust and free spaces for authentic learning.

## Introduction

How do students grow into professionals in the field of medical education? This might currently be one of the key questions in our field [1]. To consider answers to this question, we invite you to dig deep with us as we do some philosophical gardening around the idea of growth. In this paper, we explore our position as medical education professionals in relation to medical students and their development into doctors.

Gardening and medical education have in common that they both involve processes of growth and development. A gardener tends to their garden, cultivates crops, and oversees the whole process from planting seeds to ripening and harvest. A medical educator cares for their students, develops a curriculum and oversees the whole process of students turning into junior doctors. Both gardening and medical education can, as such, be seen as forms of care [2].

This exploration is philosophical because we ourselves are part of the dynamic we intend to investigate [3]. There are many ways to define philosophy, but it can be seen as making a direct connection between ways of thinking, being, doing and everyday practice. Understandably, most medical education research focuses on efficiency of educational methods for teaching, guiding and assessing medical trainees in their journey towards becoming competent health care professionals. This could be likened to examining which farming techniques, which fertilizers and which soil conditions are most effective for growing a certain crop. Instead, we focus on *the one who* applies these farming techniques, administers these fertilizers and optimizing these soil conditions. Which type of care relationship should we have to our trainees so that they grow and develop in the field of medical education?

In this paper, we use Heidegger’s work to explore the attitude that we, medical education professionals, might assume towards the process of professional growth and identity formation. We propose professional identity formation as an example of a concept that is over-theorized but pertains to a natural process that can be stimulated in practice. We distinguish three theoretical approaches in medical education literature that each conceptualize identity in a reductive way. After a reflection on identity, we point to existing educational practices that align with our view of professional identity formation.

## A tale of two gardeners

**Figure 1:**
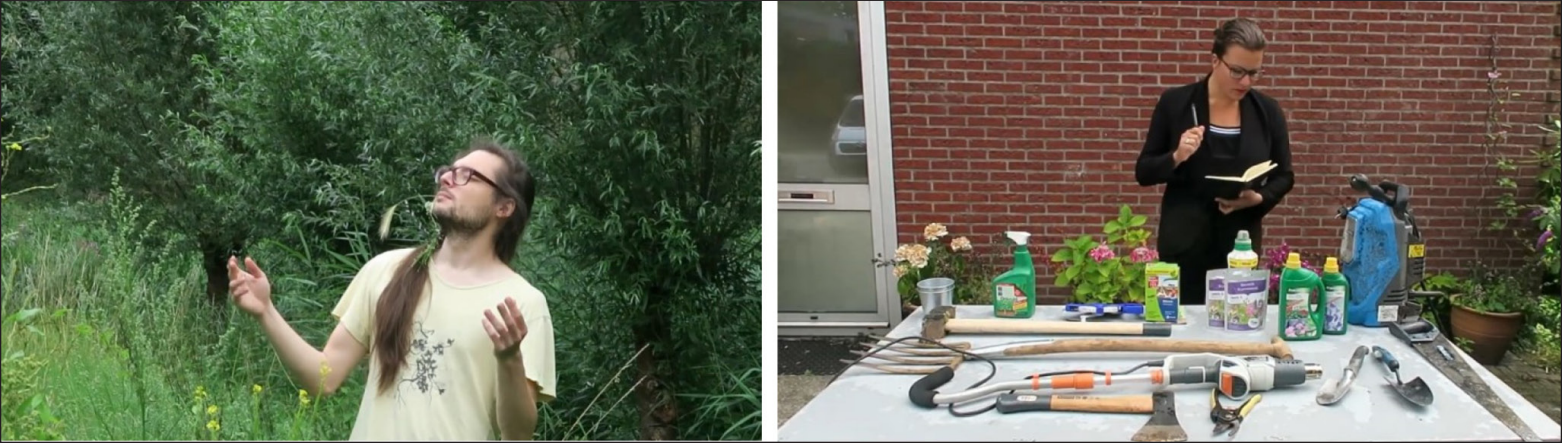
Two well-intentioned gardeners.

Please consider these two well-intentioned gardeners (See Figure 1 and video essay https://www.youtube.com/watch?v=g7jZigyfKHI) with different styles of caring for their garden. The first is an old-fashioned farmer who entrusts their seeds to the earth and watches over them, aware that the forces that make it grow – sunshine and rain, the quality of the earth – are for a large part beyond their control. Key words to this approach are cultivation, stimulation, and trust. The second takes on the approach of the modernized farming industry. Instead of entrusting the seeds to the earth, this farmer aims for maximum efficiency. This optimization requires control of every aspect of the context in which the seed grows, and predictability of the entire growing process: the chemical composition of the earth, air humidity, etc. Key words to this approach are control, reliability, and efficiency.

In *The Question After Technology*, Heidegger contrasts these two ways in which humans stimulate development:

“The field that the peasant formerly cultivated and set in order appears differently than it did when ‘to set in order’ still meant ‘to take care of’ and ‘to maintain’. The work of the peasant does not challenge the soil of the field. In the sowing of the grain he entrusts the seed to the forces that make it grow and watches over its thriving. But today, even the cultivation of the field has come under the grip of another kind of cultivation, which ‘enframes’ nature. It sets upon it in the sense of challenging it forth [4].”

Heidegger uses this example to characterize technological ways of thinking and acting. We would like to linger on the concept of ‘enframing’ which means ‘positioning’ or ‘putting in order’. Enframing describes an imperative of our modern age in which there is an emphasis on performance and efficiency, which in turn requires processes to be controllable and predictable. Heidegger uses the German word *stellen*, which can be translated as ‘bringing into position’ or ‘standing’ as in ‘standing reveille’ in the military. Similar to the way we are called upon to stand in line before boarding an airplane, the essence of *stellen* is that the soldiers or passengers position themselves in a way that makes the technical task (doing a headcount; boarding the plane efficiently) able to be performed as efficiently as possible. We can translate *stellen* as ‘presenting as’: the soldiers and passengers have to *present* themselves *as* something.

Enframing itself is not positive or negative, but simply a diagnosis of our fundamental relationship to the world around us in our age, which Heidegger calls “the age of the world picture” [5]. In medical education, we approach learning through metrics [6]. as a process in which we want to get everything ‘in the picture’: how competent a trainee is at performing a skill, or how professional or entrustable they are, for example. Heidegger uses this metaphor to point to the tendency to treat everything in terms of *performance*, an attitude that requires the subjects of care – whether crops, people or medical trainees – to ‘show up’ in a certain way in order to be seen (be represented) at all.

We feel that it is in this representational model that dominates medical education and shapes our basic approach to any issue in medical education that the problem lies. The catch-22 in representationalism is that in order to be seen as who you are, you are required to present yourself in a certain way, which by definition is something you are not (i.e., inauthenticity in the technical sense [7]). This *presenting as* and the focus of showing signs of desired qualities places trainees in a double bind. At its core, medical education is a field in which we sow seeds of physicians ‘in the bud’, with the purpose of letting them grow into competent professionals that are willing and able to fulfill medical education’s social contract with society.

The old apprenticeship model of medical education, analogous to Heidegger’s old-fashioned farmer, was characterized by a closeness to the land. This way of caring has been made sterile by newer technological ways of farming. By no means do we wish to be nostalgic though, as the newer ways of farming offer more efficient and accountable ways of training – in which the role of the doctor is ‘enframed’ in a competency framework, something that the apprenticeship model was unable to offer. But we can ask ourselves how to combine these two modes of care in a relationship to our trainees that supports a sustainable ethic for training medical professionals.

## Identity issues: what are we growing?

Becoming a physician is considered a “journey beyond competence” [8] that involves a “shift in medical education toward emphasis on the “being” as well as “doing the work” of a physician” [9]. But the question of ‘being’ and ‘becoming’ has been a topic of human inquiry for millennia. Plato already wondered if becoming is a process of growing into something new, or a remembrance of who you truly are. In medical education, becoming is usually discussed under the header of professional identity formation, “the foundational process one experiences during the transformation from lay person to physician” [10], which “involves the development of professional values, actions and aspirations and is central to medical education [11].” Using this term means we have introduced a complex concept into the field of medical education: identity.

The ubiquitous use of the term identity may lead us to believe that we know what identity is, what makes an identity professional, how identities form and what we can do to support their formation. From a philosophical perspective, however, we do not believe this to be the case. Identity is not a well-understood concept at all. In fact, it resists definition, measurement and should be seen as an open question rather than an answer. Our conception of identity is bound up with sociocultural issues of racism, gender diversity, and in general which types of identities matter and which do not [12,13,14,15]. Identity is not just about the crops, or even about the relationship between the farmer and the crops, but also about the quality of the soil – the sociocultural context – in which they grow.

Let us take a step back by first looking at how we conceptualize ‘identity’ and how we grow it. In medical education, we recognise three interrelated ways to conceptualize identity: as an individual psychological trait, as a set of behaviors, and as the way in which an individual relates to the professional community. Each of these conceptualisations is problematic in its own way, but they are all instances of the representational model of identity.

### Identity as individual trait

One view of professional identity is to see it as an individual trait. Identity is seen as located inside the trainee and that can express itself in identifiable behavior. Who you are is a ‘truth’ inside you and our challenge as educators is to mine or excavate it. Since it is fundamentally impossible to directly access another’s inner world, an individual’s public performance (the part of them that others can observe) is seen as a representation of their inner identity. Someone who has a developed professional identity acts out the behavior that is seen as professional. Professional identity is reduced to a “list of behaviors”, such as being punctual, speaking in a certain way, and projecting a certain attitude, which are taken to be “indicative” of the presence of a ‘developed’ identity: on the one hand, identity is seen as something beyond what a person says and does, but on the other hand, what a person says and does is seen as an expression of this identity. “Behaviors are […] indicative of the presence or absence of a professional identity and consequently can be used as surrogates [16].” In other words, the state of the crops is assessed by how they look, and this is taken as a sign of how nutritious they are.

### Identity as behavior

One way in which this essentialist problem is dealt with, is to reduce identity to a set of behaviors. In this behaviorist approach, behavior is not an *expression* of who you are (not an individual trait), but who you are *is* nothing but how you behave. For instance, in the case of reflection an overfocus on behavior can lead to ‘reflective zombies’: “someone who displays all the outer traits of reflection, without having actually reflected [17].” A reflective zombie is indistinguishable from a student that actually reflects from the perspective of assessment criteria that determine behavior fits the required quality. A focus on desired, ‘good’ reflection (which is represented, defined and operationalised in measurable behaviors) will lead students to show only that behavior, possibly without actually learning or reflecting. If we see behavior as a representation of inner qualities, and then reduce these inner qualities to behavior, we require a certain behavior of students. This invites students to game the system, meaning “changing behavior the moment it is assessed” [18]. This is also the case for professionalism, where quantifying professionalism make it into “just another hurdle to residency” [19], or empathy where students experience “empathic dissonance” [20] when they are required to portray behavior they do not actually feel at that time. Gaming the system is a perfect illustration of enframing: it is not a result of students’ bad intentions, but due to the focus on behavior making it more rewarding to focus on emulating the behavior that is expected rather than what it is supposed to represent. In short, it simply makes more sense to perform according to the standards, because the representation is all that matters. However, it can lead to just emulating the behavior [17], sapping internal motivation [19] and making students feel pressured and disconnected [21].

The behaviorist approach appears to solve the problem of access because it does not treat identity as a pre-existing essence that is expressed through behavior, but rather equates the behavior with identity. But denying the relevance of an inner experience related to professional identity formation mistakes the menu for the food and leads to the next problem, which is that identity is seen as *just* a performance.

### Identity as a socialization process

Identity can also be seen as a process of socialization: who you are is how you are positioned in the community. Professional identity formation is then not about an inner quality, but a process of socializing into the professional culture. This means that students learn to act in the way that their future professional culture considers ‘good practice’. A widely accepted model for socialization is the Communities of Practice model, in which novices to a field move from legitimate peripheral participation to “full participation, primarily through social interaction [21,22].” In many cases, professional identity formation is seen as an emancipatory process in which the degree to which one has formed an individual identity is precisely the degree to which one can judge when to divert from the norms and guidelines of the community. The main aspect of this does not seem to be identity, but *conformance*. In this perspective, identity is not seen as an inner quality or an objectifiable set of behavior, but rather as a social role. Here, identity becomes a performance in the sense that an actor performs a role in theatre. Depending on the play, identity can change and requires little more than a change of costume and custom.

Can a trainee adapt to the status quo? Can they *present themselves* as the image we have of a doctor? Can the trainee shape their way of talking and being to fit the dominant norms and culture of the field? In the Communities of Practice model, the need to adapt and conform seems to supersede any sense of authenticity: it does not matter if you act out your role authentically or not. This view of professional identity clashes with the idea of letting students develop in their own way, and might disallow students to be authentic [23,24]. Some researchers [25] have even suggested that this is why students with an ethnic minority get lower grades in their clinical clerkships: they do not fit the existing norm of the community of practice. Current debates about decolonizing the curriculum [26] and questioning how ‘whiteness’ [12] has been the norm in medical education painfully illustrates that the standard that the community sets is never neutral. In this sense, professional identity formation is not just about socialization, but just as much about “professional subjectification” [23] and “authenticity” [7].

### The underlying issue: representationalism

It is tempting to see these three theoretical orientations as different ways of fertilizing the field that each have their flaws, and to conclude that we need to come up with a better fertilizer, a fourth theory that includes all the benefits and none of the shortcomings. But the issue is more fundamental, that is, philosophical. In all these cases, identity is seen through the lens of representation: performance as representation of an inner world, as representation of an ideal set of behaviors, or as representation of the community standard.

Each theoretical approach emphasizes certain aspects of identity at the expense of others. However, in doing so it also *reduces* identity to that type of phenomenon [27]. A theory is a lens through which we can look at the world: identity *as* psychological state, *as* set of behaviors, *as* social role. The problem arises when *as* becomes *is*: when we say, identity *is* a psychological state (and nothing else) [28]. This is problematic because if identity is reduced to a psychological, behavioral or social process, the illusion arises that this type of research can have everything there is to know about identity ‘in the picture’. This becomes literal when we think of what theories on professional identity formation do: they present models, i.e., pictures of ‘how identity works’.

In contrast with this reductionist approach, we counter that professional identity formation has to do with the phenomenological experience of ‘what it is like’ to be a professional. So, there is an ‘inner experience’ that is relevant to identity. But whether someone is a professional or not is not just an individual choice or experience, because they are a professional in a community of skilled practitioners. Professional identity formation is a great name for this process. However, in relation to becoming the doctor that *you* (the trainee) are, *we* (the medical education professional) should ask ourselves:

– Can we ever claim to know who you really are, as a (trainee) doctor?
– Can we show you a model or a pathway for how to become who you are?
– Can we support you in the process of becoming who you are?

## Ecological diversity

Theoretical approaches to identity can give us the comforting feeling that we know what identity is and that we can even ‘manage’ it. But we can learn something from the integration of sustainable farming approaches and the efficiency and performance of modern approaches.

Uniformity, predictability and conformity lead to loss of biodiversity, while ecologically diverse growing increases biodiversity while taking care of the soil. Rather than a one size fits all approach, we should stimulate small-scale experiments that cross-fertilize each other without having to conform to one approach. This also means improvising and figuring out what works in the process, rather than presenting a detailed plan and then executing it (which is a requirement from the perspective of enframing).

The second lesson is that instead asking what we can ‘do’ to remedy loss of internal motivation, authentic reflection or the feeling of connection, is that as a recent study suggests, tropical forests may regrow most effectively when left alone [29]. This does not necessarily mean that we should ignore identity, professionalism, reflection, empathy and so on. Rather, we can consider how we can give trainees more space with time to reflect on their own experience and perspective, and opportunity to explore other perspectives. While we do not have definite answers – if we did, we would ourselves cotradict our proposed approach – we do feel that there might be some necessary ingredients to make PIF part of the curriculum in a sustainable way. The first is respecting autonomy of the student. Students are allowed to be different, and are invited to think about what complex concepts mean to them. They are held responsible for their own choices, and are seen as individuals. The second ingredient is to take step away from the modus operandi– sometimes literally taking students to a different location – and show them different feeding ground. This is a prime example of what Kumagai & Wear call ‘making strange’ [30] (a core philosophical practice [31]), to help disrupting and disturbing ‘one’s assumptions, perspectives, and ways of acting so that one sees the self, others, and the world anew’ [32]. Other examples of ‘making strange’ initiatives are letting students write love and break up letters to empathy [33], using Harry Potter stories to learn about empathy [34], and Medical Education Empowered by Theatre (MEET) uses theatre in which the audience (i.e. the students) can modulate the characters’ attitudes, behaviors, or personalities [35]. The third ingredient of best practice initiatives is organizing human contact in medical training. After all, *personal* growth and development require a *personal* approach. Mentoring is a good example when it comes to educator-student contact: it requires the mentor to really get to know students. Another one is to provide students with opportunities to interact with patients early on in the medical curriculum, instead of “representations of patients” [32,36].

An overarching result from all these good practices is that they invest time in the design of meaningful experiences, and in tailoring guidance and facilitation of learning. They focus less (or not at all) on testing and grading students – which are activities prone to evoke a spirit of representationalism. They prioritize stimulating growth over measuring it.

## Growing our field

We have attempted to take a philosophical approach to our relationship to our trainees. We chose professional identity formation as a focus because how we conceptualize the identity of our trainees shapes our relationship to them. But we cannot describe the phenomenon of professional identity formation from a critical distance because it is too close to us. After all, *we* are also working on our professional identity as members of the medical education community, in teacher training and in journals such as this one. We cannot occupy a neutral or objective vantage point outside of the cultural, historical and professional context that determines what is understood by professional growth at any given time. Any theoretical description of professional growth might be seen as a performance of our own values which, as we know, are a mirror of our own approach rather than a property of the phenomenon we aim to theorize.

The old-fashioned farmer in Heidegger’s essay about ‘enframing’ [4] focuses on providing them with what they require at every moment, and taking away barriers to growing into their full potential. The modern farmer, instead, focuses on how to make the crops behave according to a model (picture) of what the crops should look like once they are grown. Which is better? The reality is, we do not have this choice. We cannot (and should not want to) return to the apprenticeship model, and we want to maintain the benefits of our modern approach.

Just like farming in the context of the climate crisis, we realize that a focus on growth and efficiency is not sustainable, but at the same time to feed the world’s population (or provide it with health care professionals), the old ways of farming are unable to provide what contemporary society requires. So, we do need growth, but in another sense. Current concepts of professional identity formation no longer meet the needs of contemporary medical education.

In this paper, we have focused on identity as a core concept that shapes our relationship with trainees. But medical education has recently seen initiatives that fundamentally interrogate other complex concepts that that we use every day: reflection [37], professionalism [38][15], care [2], science [39], technology [40], and skillification [41]. Many of these concepts are ways to create a conscious relationship to our field, within which metrics and technical thinking have their proper place. Nimmon formulated this dilemma as “the tension between our desire to maintain a sense of humanism among our practitioners and our teaching processes that promote the technification of medical expertise” [42]. Her concept of technification can be seen as an aspect of representationalism: “the repurposing of human social activity to function as a procedural or diagnostic skill designed to gather data about the patient and determine what aspect of the patient requires curing.” [42] But how can we expect students and trainees to resist technification of the humane side of medical practice if we ourselves technify medical education?

We need growth in the sense of the development of the concepts that we use to shape our relationship to our trainees. These concepts need to be precise, without being too rigid. The way to do this is to keep them in a dialogue with practice, in which they can continue to be sharpened by the reality of the classroom and residency, while also providing educational practice with guidance on how to design it. This interplay between practice, theory and concepts is what we call philosophical gardening: working while thinking, thinking while working.
